# Contamination of *Streptococcus suis* and *S. suis* Serotype 2 in Raw Pork and Edible Pig Organs: A Public Health Concern in Chiang Mai, Thailand

**DOI:** 10.3390/foods13132119

**Published:** 2024-07-03

**Authors:** Ratchadakorn Guntala, Likhitphorn Khamai, Nattawara Srisai, Sakaewan Ounjaijean, Woottichai Khamduang, Sayamon Hongjaisee

**Affiliations:** 1 School of Health Sciences Research, Research Institute for Health Sciences, Chiang Mai University, Chiang Mai 50200, Thailand; ratchadakorn_g@cmu.ac.th (R.G.); sakaewan.o@cmu.ac.th (S.O.); 2 Research Institute for Health Sciences, Chiang Mai University, Chiang Mai 50200, Thailand; likhit.parinn@gmail.com; 3 Department of Medical Technology, Faculty of Associated Medical Sciences, Chiang Mai University, Chiang Mai 50200, Thailand; lookwawang@hotmail.com (N.S.); woottichai.k@cmu.ac.th (W.K.); 4 LUCENT International Collaboration, Faculty of Associated Medical Sciences, Chiang Mai University, Chiang Mai 50200, Thailand

**Keywords:** *Streptococcus suis*, *Streptococcus suis* serotype 2, foodborne pathogens, Thailand, raw pork meat

## Abstract

*Streptococcus suis* is one of the most important zoonotic pathogens causing serious diseases in both pigs and humans, especially serotype 2. In northern Thailand, there is a notable prevalence of *S. suis* infection in humans and transmission has occurred mainly through the consumption of raw pork products. Despite the continued practice of consuming raw pork in this region, limited data exist regarding *S. suis* contamination in such products. Therefore, this study aimed to assess the prevalence of *S. suis* and *S. suis* serotype 2 in retail raw pork meat and edible pig organs sold in Chiang Mai city, Thailand. A total of 200 samples, comprising raw pork meat and edible pig organs, were collected from nine fresh markets in Chiang Mai city between May and July 2023. Samples were prepared and cultured in Todd-Hewitt broth. Bacterial DNA was extracted and tested for any serotypes of *S. suis* and serotype 2 using loop-mediated isothermal amplification (LAMP) techniques. The study revealed contaminations of *S. suis* and serotype 2 at rates of 84% and 34%, respectively, with a higher prevalence observed in pig organs compared to raw pork. Both *S. suis* and serotype 2 were detected across all nine fresh markets investigated. The prevalence of *S. suis* remained consistently high throughout the study period, whereas serotype 2 showed peaks in May and July. These high rates of contamination indicate that people who consume or work in close contact with raw pork or edible pig organs are at a high risk of *S. suis* infection. Urgent implementation and maintenance of food safety campaigns and public health interventions are crucial for disease prevention and control.

## 1. Introduction 

*Streptococcus suis* is a Gram-positive bacterium that is one of the most important pathogens, causing meningitis, arthritis, and septicemia primarily in pigs, and particularly affecting piglets. It is considered a zoonotic disease from animals to humans. *S. suis* infection in humans typically occurs through skin wounds when handling infected pigs, during food preparation, or through the consumption of raw pork or fresh blood contaminated with the bacteria [1]. In humans, *S. suis* infection can lead to meningitis, arthritis, endocarditis, septicemia, and hearing loss in severe cases, or even death [2]. The majority of reported human cases globally, approximately 80%, originate from China, Thailand, and Vietnam, with serotype 2 accounting for about 75% of these cases [1]. The high prevalence of *S. suis* infection in East Asian and Southeast Asian countries may relate to factors including dense pig populations, numerous pig farms, few preventive measures during slaughtering, the presence of wet markets, and the consumption of raw pork. Data from the Bureau of Epidemiology, Department of Disease Control, Thai Ministry of Public Health, indicated an increasing trend in human cases of *S. suis* infection from 2011 to 2021, with *S. suis* serotype 2 being predominantly isolated [3]. Recently, in 2023, a total of 548 cases of infection with 26 deaths were reported in several provinces, especially in northern Thailand. A previous report estimated an incidence rate of 6.2 per 100,000 in the general population in Phayao province, with a high fatality rate of 16%, and the transmission occurred mainly through the consumption of raw pork products [4]. At least five outbreaks have been documented in Thailand; four of these were reported in the north and have been related to the consumption of raw pork/pig’s blood dishes [3]. 

Diagnosis of *S. suis* is critical for effective patient management, treatment, and disease control. Laboratory diagnosis of *S. suis* infection traditionally relies on bacterial culture and biochemical testing, but the process is time-consuming, taking at least 3 days to provide the results. There is also a risk of misidentification with other bacteria due to potential ambiguity in biochemical results [5,6]. To overcome this limitation, molecular techniques specific to *S. suis* or *S. suis* serotype 2 including polymerase chain reaction (PCR), multiplex PCR, and real-time PCR have also been developed [7,8,9,10,11], offering higher sensitivity and specificity but requiring expensive equipment and specialized skills. The loop-mediated isothermal amplification assay (LAMP) has emerged as a preferred alternative method due to its simplicity, rapidity, user-friendliness, and lower cost. LAMP can be performed at a constant temperature (60–65 °C) for 30–60 min and allows for visual detection of results through turbidity or color change, making it accessible even without specialized equipment. Several studies have successfully developed the LAMP technique to detect *S. suis* or *S. suis* serotype 2 with high specificity and sensitivity [12,13,14,15,16]. Some studies applied the LAMP technique to detect *S. suis* and *S. suis* serotype 2 from raw pork meat samples, suggesting its potential as a valuable tool for surveillance in market settings [12,17]. A study conducted in the central region of Thailand also used the LAMP technique to detect *S. suis* and *S. suis* serotype 2 from pork samples and reported very high contamination of *S. suis* and *S. suis* serotype 2 in raw pork and edible pig organs, with *S. suis* detected in up to 85% of samples and serotype 2 in 17% [17], underscoring the potential risk posed by raw pork or edible pig organs as sources of human infection.

Several studies also reported human cases with *S. suis* infection in the northern region and have been related to the consumption of raw pork products [4,18,19,20,21,22]. Despite the known risks, the consumption of raw pork persists among certain populations, increasing the likelihood of exposure to infectious bacteria. However, comprehensive data on the contamination of *S. suis* in raw pork remains limited. Therefore, this study aimed to assess the prevalences of *S. suis* and *S. suis* serotype 2 in retail raw pork meat and edible pig organs sold in Chiang Mai city, Thailand.

## 2. Materials and Methods

### 2.1. Sample Collection and Preparation

**Figure 1:**
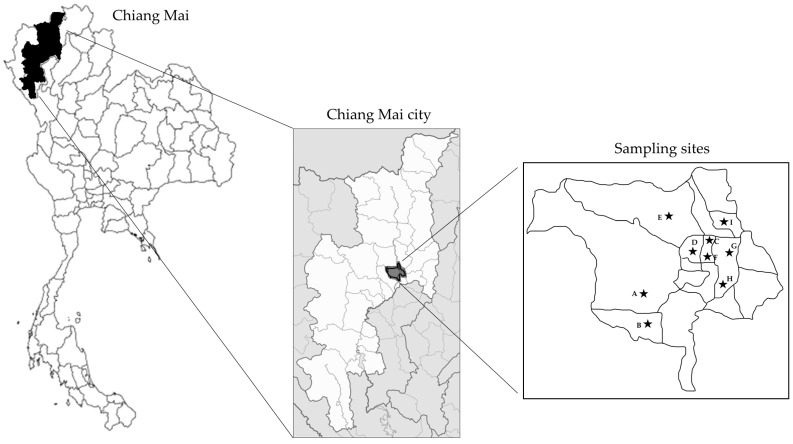
Locations of the sites. The black stars on the map indicate locations of the 9 fresh markets (Market A–I) in Chiang Mai city.

A total of 200 pork meat samples, consisting of 170 raw pork samples and 30 edible pig organ samples (20 hearts and 10 lungs) were collected from 9 fresh markets located in Chiang Mai city from May to July 2023 (Figure 1). The raw pork was a pork tenderloin, a long, narrow, boneless cut of meat that comes from the muscle and runs along the backbone. This part is popular for cooking “Larb”, which is a northern Thai dish commonly eaten in raw form in northern Thailand. We randomly collected approximately 20 samples per week. Each sample was chosen from a different stall. Raw pork samples were immediately transported to examine in the laboratory on the same day after purchasing. Samples were prepared according to the protocol of the previous study [12] with slight modifications. Briefly, samples were cut into small pieces with a total of 100 g and then put in a zip-lock bag. Thirty mL of saline solution was added to the sample and mixed by a Stomacher machine or blender. Then, 100 µL was added to 5 mL Todd-Hewitt broth containing Streptococcus Selective Supplement (HiMedia, Mumbai, India) and incubated at 37 °C for 18–20 h.

### 2.2. Bacterial DNA Extraction

After incubation, 2 mL of bacterial culture was centrifuged at 4000× *g* for 10 min to obtain the cell pellet. Bacterial DNA was then extracted using the E.Z.N.A.^®^ Bacterial DNA Kit (Omega Bio-tek, Inc., Norcross, GA 30071, USA), according to the manufacturer’s instruction. Briefly, a bacterial cell lysate was prepared by adding 100 µL of TE buffer and 10 µL of Lysozyme, which were incubated at 37 °C for 10 min. Then, 100 µL of TL buffer and 20 µL of Proteinase K were added and incubated at 55 °C for 60 min and subsequently mixed with 5 µL of RNase A. A supernatant was collected after centrifuging at 10,000× *g* for 2 min, and then mixed with 220 µL of BL buffer and 220 µL of 100% ethanol. Subsequently, binding, washing, and elution were performed to obtain the purified DNA. Between each step, centrifugation was performed at 10,000× *g* for 1 min. The total amount and purity of bacterial DNA were determined by spectrometry at 260 and 280 nm in a NanoDrop spectrophotometer (Thermo Scientific™, Waltham, MA, USA) and then stored at −80 °C freezer until being tested for *S. suis* contamination.

### 2.3. Detections of S. suis and S. suis Serotype 2 by LAMP Techniques 

The DNA extracts from meat sample culture were tested for the presences of *S. suis* and *S. suis* serotype 2 using LAMP techniques, specifically LAMP-SS and LAMP-SS2, respectively. The LAMP-SS technique, used to detect *S. suis*, involved five primers: forward outer primer (F3), backward outer primer (B3), forward inner primer containing F1 linked F2 (FIP), backward inner primer containing B1 linked B2 (BIP), and loop backward primer (LB), which targets the *S. suis recN* gene as described in the study by Arai et al. [12]. Samples positive for *S. suis* were further tested for *S. suis* serotype 2 using LAMP-SS2. The LAMP-SS2 technique also utilized five primers: F3, B3, FIP, BIP, and LB, which target the *cps2J* gene specific to *S. suis* serotype 2, according to the study by Zhang et al. [13]. 

For both LAMP-SS and LAMP-SS2, a 25 µL reaction mixture was prepared, containing 10 mM (NH_4_)_2_SO_4_, 50 mM KCl, 0.1% *v*/*v* Tween-20, 8 mM MgSO_4_, and 1.4 mM dNTP Mix. Then, 1.6 µM of FIP and BIP primers, 0.2 µM of F3 and B3 primers, 0.8 µM LB primer, 8 U *Bst* DNA Polymerase (New England Biolabs, Ipswich, MA, USA), and 2 µL of DNA template were added into the reaction mixture. Sterile water was added to bring the total volume to 25 µL. Additionally, 0.8 mM Betaine was added to the LAMP-SS2 reaction to enhance the efficiency of the amplification process [13]. LAMP-SS and LAMP-SS2 were performed at 65 °C for 45 min and then inactivated at 80 °C for 2 min.

To facilitate result interpretation, 100 µM cresol red (Sigma-Aldrich, St. Louis, MO, USA) was pre-added to the LAMP-SS and LAMP-SS2 mixtures as a visual indicator dye to measure pH change during the amplification process. During DNA amplification, the DNA polymerase incorporates deoxynucleotide triphosphate into the nascent DNA, releasing by-products including pyrophosphate and hydrogen ions, leading to a drop in pH. The initial pH of the LAMP mixture is approximately 8.8–9.0, and after amplification, it typically drops to 6.0–6.5 [23]. This pH drop causes the cresol red dye to change color from pink to yellow. A positive result is indicated by a color change from pink to yellow, while a negative result retains the pink color. To confirm the results, 5 µL of the LAMP product was subjected to 2% agarose gel electrophoresis with RedSafe dye (iNtRON Biotechnology in Gyeonggi do, Republic of Korea) under UV irradiation. Positive LAMP products appeared on the stained gel as a typical ladder pattern with multiple bands of different sizes.

### 2.4. Statistical Analysis

The prevalence of *S. suis* was presented with percentages and a 95% confidence interval (CI). The prevalence of *S. suis* was defined as the number of pork samples testing LAMP positive divided by the total number of pork samples tested. The Chi square test was used to test whether there were any significant differences in positive samples between raw pork and edible organs, between the 9 markets, and between the months of sample collection. All data were analyzed using Stata version 16.0 software (StataCorp, College Station, TX, USA). A *p*-value < 0.05 was considered as a statistically significant difference.

## 3. Results

### 3.1. LAMP Results for S. suis and S. suis Serotype 2 Detection

**Figure 2:**
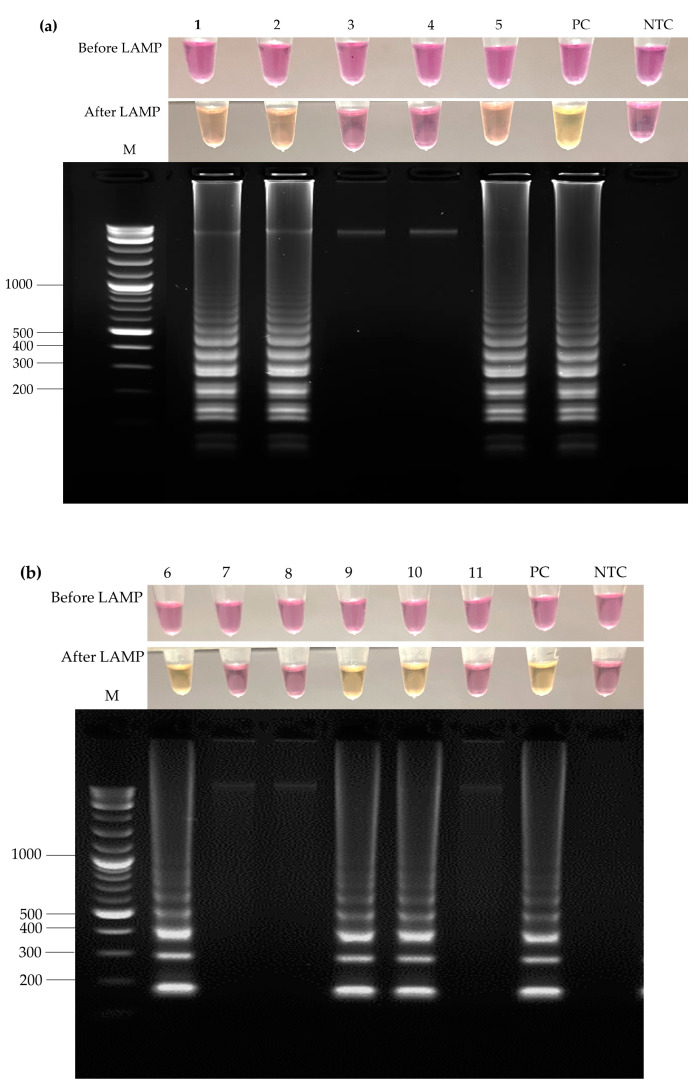
LAMP results for *Streptococcus suis* and *Streptococcus suis* serotype 2 detection in the first 11 representative raw pork samples. Lanes 1–11 represent the raw pork samples. (**a**) Results from LAMP-SS targeting *S. suis*, three samples were positive (lanes 1, 2, and 5) and two samples were negative (lanes 3 and 4). (**b**) Results from LAMP-SS2 targeting *S. suis* serotype 2, three samples were positive (lanes 6, 9, and 10) and three samples were negative (lanes 7, 8, and 11). DNA extracted from *S. suis* serotype 2 was used as positive control in both LAMP-SS and LAMP-SS2. The pattern of the LAMP products on gel electrophoresis corresponded with color change in the reaction tube. The maker (M) was a 1 kb DNA ladder. PC, positive control; NTC, no-template control.

The colorimetric LAMP techniques revealed a color change from pink to yellow in the reaction mixture for tubes containing *S. suis* (Figure 2a) and *S. suis* serotype 2 (Figure 2b). All samples were also confirmed by gel electrophoresis. Positive samples showed the LAMP product on the stained gel as a characteristic ladder DNA pattern, while no bands were detected in the no-template control and negative samples.

In addition, we tested the performance of the colorimetric LAMP-SS and LAMP-SS2; the limits of detection of the LAMP-SS and LAMP-SS2 techniques were 0.01 and 0.1 ng/µL, respectively. Cross-reactivity was not found when tested with 17 other bacterial strains including *Streptococcus pyogenes*, *Streptococcus agalactiae*, *Streptococcus pneumoniae*, *Streptococcus viridans*, *Staphylococcus aureus*, *Haemophilus influenzae*, *Acinetobacter baumannii*, *Klebsiella pneuminiae*, *Escherichia coli*, *Enterobacter aerogenes*, *Enterococcus faecalis*, *Salmonella enteritidis*, *Streptococcus bovis*, *Streptococcus oralis*, *Streptococcus mutans*, *Pseudomonas aeruginosa*, and *Niesseria meningitidis*.

### 3.2. Prevalence of S. suis and S. suis Serotype 2 Contamination in Raw Pork and Edible Pig Organs 

Among the 200 samples tested, 167 samples were positive for *S. suis* (84%, 95% CI: 78–88%), and 68 samples were positive for *S. suis* serotype 2 (34%, 95% CI: 28–41%).

**Figure 3:**
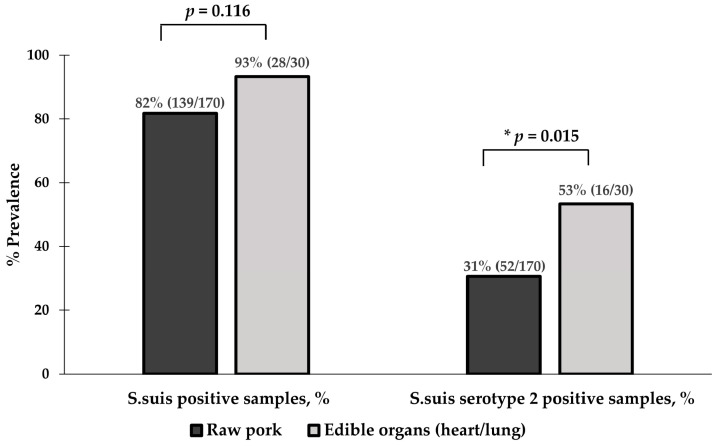
Prevalence of *S. suis* and *S. suis* serotype 2 contamination based on sample type. * indicates a statistically significant result (*p* < 0.05).

Regarding sample type (Figure 3), the contamination rate of *S. suis* in edible pig organs (heart and lung) was higher than in raw pork (93% vs. 82%, *p* = 0.116), but it was not statistically significant. The contamination rate of *S. suis* serotype 2 in edible pig organs was significantly higher than in raw pork (53% vs. 31%, *p* = 0.015). The 30 edible pig organs consisted of 20 hearts and 10 lungs. The prevalence rate of *S. suis* was 100% in lung samples (10/10) and 90% in heart samples (18/20). For *S. suis* serotype 2, the prevalence rate was 60% in heart samples (12/20) and 40% in lung samples (4/10). 

### 3.3. Prevalence of S. suis and S. suis Serotype 2 Contamination from Fresh Markets

**Figure 4:**
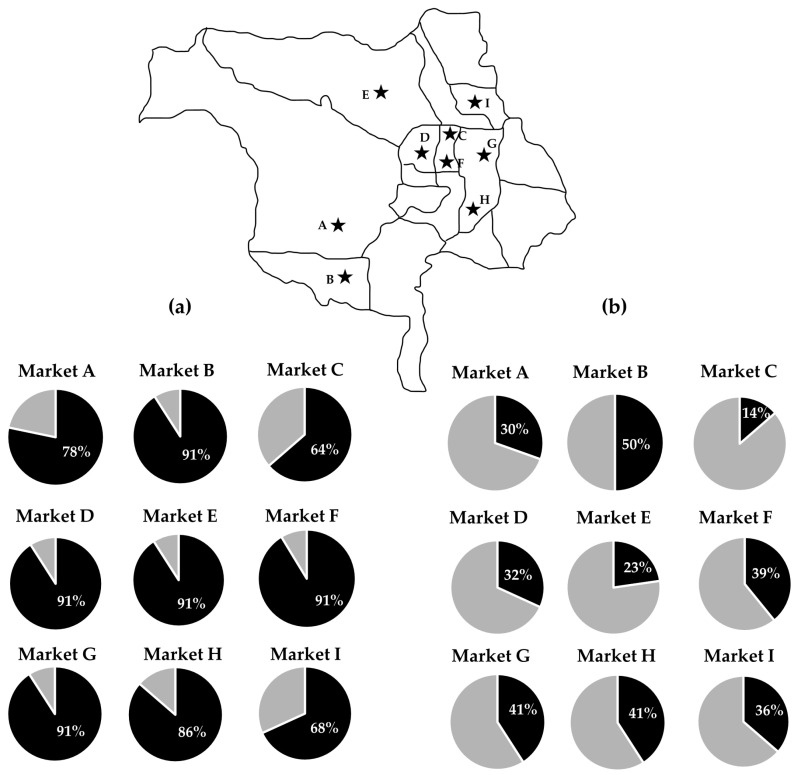
Pie charts represent the prevalences of *S. suis* (**a**) and *S. suis* serotype 2 (**b**) contamination among 9 fresh markets located in Chiang Mai city, Thailand. Black area in the pie chart represents the percent of positive samples. The black stars on the map indicate locations of the 9 fresh markets (Market A–I) in Chiang Mai city.

Regarding the sample sources (Figure 4), *S. suis* and *S. suis* serotype 2 were found in all nine fresh markets investigated in this study with varying prevalence rates. The prevalence of *S. suis* contamination ranged from 64% to 91%. The highest prevalent rates of *S. suis* positive samples were found in market B (20/22, 91%), market D (20/22, 91%), market E (20/22, 91%), market F (21/23, 91%), and market G (20/22, 91%), followed by market H (19/22, 86%), market A (18/23, 78%), market I (15/22, 68%), and market C (14/22, 64%). However, there was no statistically significant difference between the markets (*p* = 0.056).

The prevalence of *S. suis* serotype 2 contamination ranged from 14 to 50%. The highest prevalent rates of *S. suis* serotype 2 positive samples were found in market B (11/22, 50%), followed by market G (9/22, 41%), market H (9/22, 41%), market F (9/23, 39%), market I (8/22, 36%), market D (7/22, 32%), market A (7/23, 30%), market E (5/22, 23%), and market C (3/22, 14%). However, these differences were not statistically significant (*p* = 0.321).

### 3.4. Prevalence of S. suis and S. suis Serotype 2 Contamination According to the Month of Sample Collection

**Figure 5:**
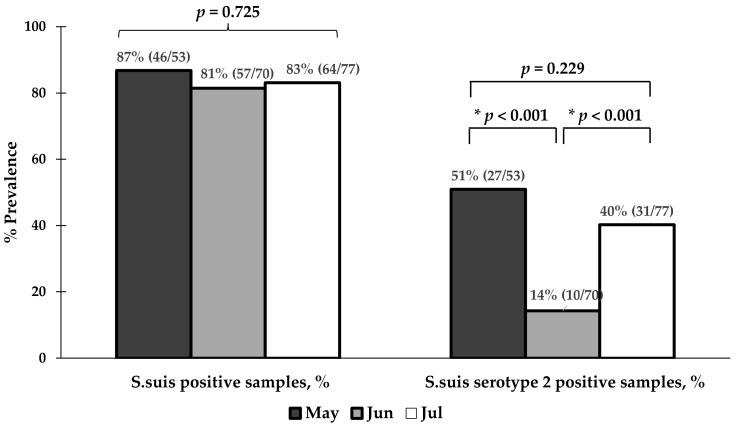
The prevalence rates of *S. suis* and *S. suis* serotype 2 contamination in raw pork/edible pig organs collected from 9 fresh markets according to the month of sample collection. * indicates a statistically significant result (*p* < 0.05).

In addition, we analyzed the prevalences of *S. suis* and *S. suis* serotype 2 contamination in raw pork and edible pig organs collected from nine fresh markets based on the month of sample collection (Figure 5). The results showed that the prevalence of *S. suis* contamination remained stable throughout the study period (87% in May, 81% in June, and 83% in July) with no statistically significant difference (*p* = 0.725). In contrast, the prevalent rate of *S. suis* serotype 2 varied over the sample collection period. As shown in Figure 5, the prevalence of *S. suis* serotype 2 positive samples in June were significantly lower than in May (14% vs. 51%, *p* < 0.001) and July (14% vs. 40%, *p* < 0.001).

## 4. Discussion

In this study, we found *S. suis* and *S. suis* serotype 2 in both edible pig organs and raw pork collected from nine fresh markets in Chiang Mai from May to July 2023, with high contamination rates of 84% and 34%, respectively. Our results indicated that the contamination rate of *S. suis* in edible pig organs was higher than in raw pork, especially for *S. suis* serotype 2.

The prevalence rates of *S. suis* and *S. suis* serotype 2 in this study were higher than those reported in previous studies in Thailand [17,24]. Noppon et al., 2014 reported a prevalence of 12.8% for *S. suis* serotype 2 in 320 uncooked pork samples in northeastern Thailand using a multiplex PCR assay [24]. They also found a significant contamination rate of 24.6% in fresh pig blood samples. Additionally, a study in central Thailand showed high contamination rates of *S. suis* and *S. suis* serotype 2 in 88 raw pork samples, with rates of 85.2% and 17.1%, respectively, using LAMP techniques [17]. Although the prevalence of *S. suis* in that study was similar to our study, the prevalence of *S. suis* serotype 2 was lower, possibly due to the smaller sample size tested. Variations in the prevalence of *S. suis* and *S. suis* serotype 2 might be due to the differences in sample collection methods, the number of samples tested, detection techniques, locations, and study periods. 

Although the natural habitat of *S. suis* is the tonsil and nasal cavities of pigs, it can also be isolated from various organs of diseased pigs such as the lung, brain, joint fluid, blood, spleen, vaginal swab, pleural effusion, and tongue swab [25]. Consequently, we expected organ samples to be contaminated with *S. suis*. Previous studies have also detected *S. suis* and *S. suis* serotype 2 in organs such as the liver, lung, heart, kidney, and brain [17,25,26]. In our study, the prevalence rate of *S. suis* and *S. suis* serotype 2 contamination was higher in organs (heart and lung) compared to raw pork samples. This suggests that after penetration of host mucosal barriers, *S. suis* can reach and survive in the blood and then invade multiple organs, including the lung, spleen, liver, kidney, heart, and brain [27]. The ability of *S. suis* to invade multiple organs results in higher contamination rates in these organs compared to pork meat. Moreover, cross-contamination between pig organs and meat may occur through meat cutting or handling during slaughtering or post-slaughter processes. These findings suggested that pig organs are major targets for *S. suis* infection and may be a significant source of *S. suis* and *S. suis* serotype 2 in human infections.

In the nine fresh markets investigated in this study, contamination of *S. suis* and *S. suis* serotype 2 was found in raw pork in all markets. These nine markets, located in Chiang Mai city, sell a variety of fresh products including fruits, vegetables, pork, beef, and other foods. The high contamination rate may be due to poor sanitation and hygiene practices in these local markets. Some practices, such as working without gloves, using shared utensils like chopping boards, knives, or buckets with all meat types, can lead to cross-contamination with contaminated meat. The environmental conditions in markets, such as temperature and humidity, can also further exacerbate the issue. Poor sanitation and hygiene practices coupled with environmental conditions can enable the growth and spread of *S. suis*, leading to a higher contamination rate. Additionally, the pork supply chain from the slaughterhouse to retail markets may contribute to high contamination rates during slaughtering, butchering, processing, or distribution. Screening for *S. suis* at the slaughterhouse, either ante-mortem or post-mortem, may be necessary to ensure meat safety and control the disease. 

Based on the study period, the samples were collected from May to July, covering the end of summer and the beginning of the rainy season. Our results showed that the prevalence of *S. suis* contamination was high and stable during this period, but the prevalence of *S. suis* serotype 2 positive sample was higher in May and July compared to June. This is consistent with a previous study in Thailand indicating a high incidence of *S. suis* disease during the rainy season (June–September) [28]. A retrospective study reported a high number of *S. suis* cases admitted to Chiang Mai University Hospital during summer (April–June) and the rainy season (August–October) [29]. Another study in Phayao province showed peak incidence in May [4]. Hot and humid weather in these seasons may increase stress on pigs during transportation, leading to a higher occurrence of infection. In addition, these conditions may enable the organism to proliferate and increase infectivity during meat exposure [30]. These findings suggested that raw pork may be a source of disease in humans, especially since consuming raw pork is still a common practice in northern Thailand. Thus, food safety measures must be emphasized. 

In this study, LAMP techniques were used to investigate the prevalence of *S. suis* and *S. suis* serotype 2 contamination in raw pork from fresh markets in Chiang Mai city. Previous studies also used LAMP techniques for detection, with results observed by turbidity or gel electrophoresis [12,17]. In this study, we used colorimetric LAMP based on cresol red indicator dye, which is simple, rapid, and allows easy observation of results through a color change. This technique is beneficial as a surveillance tool for detection of *S. suis* contamination from environmental samples. Several studies have demonstrated that LAMP techniques can be used for pathogen detection in environmental samples such as soil, water, fruits, or vegetables [31,32,33,34]. 

This study has some limitations, including a relatively small sample size and a short observation period. Not all types of pig organs were collected. However, this study demonstrated the occurrence of *S. suis* and *S. suis* serotype 2 in raw pork and edible pig organs in Chiang Mai city, northern Thailand. Further studies would benefit from large sample sizes and expanded sampling areas across other provinces or regions of the country. 

Due to the high prevalence of *S. suis* and *S. suis* serotype 2 contamination, individuals who work closely with raw pork, especially butchers, may be at high risk for *S. suis* infection. Consuming raw pork or partially cooked pork products is also a high-risk factor for *S. suis* infection. Although, a previous study showed the effectiveness of the food safety campaign in controlling the *S. suis* infection in humans in Thailand [35], new cases still occur annually, especially in the northern region, indicating persistent cultural practices. Changing consumption habits may be difficult due to the cultural and societal factors involved [36]. Therefore, a food safety campaign and public health intervention are needed to implement and maintain prevention and control of the disease. A combination of measures to prevent disease transmission should be focused on and implemented in communities.

## 5. Conclusions

In conclusion, we demonstrated a high prevalence of *S. suis* and *S. suis* serotype 2 in raw pork and edible pig organs collected from nine fresh markets in Chiang Mai city, Thailand. Individuals involved in the pig industry, including those working on pig farms or slaughterhouses, and those who consume raw pork should be aware of the potential hazards of *S. suis* infection. Continuous education through food safety campaigns and alternative public health interventions are essential to prevent, control, and eliminate the disease. 

## Data Availability

The original contributions presented in the study are included in the article; further inquiries can be directed to the corresponding author.

## References

[1] Goyette-Desjardins G., Auger J.P., Xu J., Segura M., Gottschalk M. (2014). *Streptococcus suis*, an important pig pathogen and emerging zoonotic agent-an update on the worldwide distribution based on serotyping and sequence typing. Emerg. Microbes Infect..

[2] Huong V.T., Ha N., Huy N.T., Horby P., Nghia H.D., Thiem V.D., Zhu X., Hoa N.T., Hien T.T., Zamora J. (2014). Epidemiology, clinical manifestations, and outcomes of *Streptococcus suis* infection in humans. Emerg. Infect. Dis..

[3] Kerdsin A. (2022). Human *Streptococcus suis* Infections in Thailand: Epidemiology, Clinical Features, Genotypes, and Susceptibility. Trop. Med. Infect. Dis..

[4] Takeuchi D., Kerdsin A., Pienpringam A., Loetthong P., Samerchea S., Luangsuk P., Khamisara K., Wongwan N., Areeratana P., Chiranairadul P. (2012). Population-based study of *Streptococcus suis* infection in humans in Phayao Province in northern Thailand. PLoS ONE.

[5] Palmieri C., Varaldo P.E., Facinelli B. (2011). *Streptococcus suis*, an Emerging Drug-Resistant Animal and Human Pathogen. Front. Microbiol..

[6] Tarradas C., Arenas A., Maldonado A., Luque I., Miranda A., Perea A. (1994). Identification of *Streptococcus suis* isolated from swine: Proposal for biochemical parameters. J. Clin. Microbiol..

[7] Hatrongjit R., Kerdsin A., Gottschalk M., Hamada S., Oishi K., Akeda Y. (2016). Development of a multiplex PCR assay to detect the major clonal complexes of *Streptococcus suis* relevant to human infection. J. Med. Microbiol..

[8] Ishida S., Tienle H.T., Osawa R., Tohya M., Nomoto R., Kawamura Y., Takahashi T., Kikuchi N., Kikuchi K., Sekizaki T. (2014). Development of an appropriate PCR system for the reclassification of *Streptococcus suis*. J. Microbiol. Methods.

[9] Kerdsin A., Akeda Y., Hatrongjit R., Detchawna U., Sekizaki T., Hamada S., Gottschalk M., Oishi K. (2014). *Streptococcus suis* serotyping by a new multiplex PCR. J. Med. Microbiol..

[10] Marois C., Bougeard S., Gottschalk M., Kobisch M. (2004). Multiplex PCR assay for detection of *Streptococcus suis* species and serotypes 2 and 1/2 in tonsils of live and dead pigs. J. Clin. Microbiol..

[11] Goto Y., Fukunari K., Tada S., Ichimura S., Chiba Y., Suzuki T. (2023). A multiplex real-time RT-PCR system to simultaneously diagnose 16 pathogens associated with swine respiratory disease. J. Appl. Microbiol..

[12] Arai S., Tohya M., Yamada R., Osawa R., Nomoto R., Kawamura Y., Sekizaki T. (2015). Development of loop-mediated isothermal amplification to detect *Streptococcus suis* and its application to retail pork meat in Japan. Int. J. Food Microbiol..

[13] Zhang J., Zhu J., Ren H., Zhu S., Zhao P., Zhang F., Lv H., Hu D., Hao L., Geng M. (2013). Rapid visual detection of highly pathogenic *Streptococcus suis* serotype 2 isolates by use of loop-mediated isothermal amplification. J. Clin. Microbiol..

[14] Hess J., Kreitlow A., Rohn K., Hennig-Pauka I., Abdulmawjood A. (2023). Rapid Diagnostic of *Streptococcus suis* in Necropsy Samples of Pigs by thrA-Based Loop-Mediated Isothermal Amplification Assay. Microorganisms.

[15] Meng J., Li C., Wang Y., Bian Z., Chu P., Zhai S., Yang D., Song S., Li Y., Jiang Z. (2022). Accelerated loop-mediated isothermal amplification method for the rapid detection of *Streptococcus suis* serotypes 2 and 14 based on single nucleotide polymorphisms. Front. Cell. Infect. Microbiol..

[16] Li L., Ren J., Zhang Q., Luo Y., Zhang Y., Qi J., Zhao X., Hu M., Liu Y. (2022). Development of Two Loop-Mediated Isothermal Amplification Assays for Rapid Detection of ermB and mefA Genes in *Streptococcus suis*. Foodborne Pathog. Dis..

[17] Boonyong N., Kaewmongkol S., Khunbutsri D., Satchasataporn K., Meekhanon N. (2019). Contamination of *Streptococcus suis* in pork and edible pig organs in central Thailand. Vet. World.

[18] Fongcom A., Pruksakorn S., Mongkol R., Tharavichitkul P., Yoonim N. (2001). *Streptococcus suis* infection in northern Thailand. J. Med. Assoc. Thail. Chotmaihet Thangphaet.

[19] Khadthasrima N., Hannwong T., Thammawitjaya P., Pingsusean D., Akkanij B., Jaikhar A., Paungmali P., Yudee P., Wongyai S., Samerchea S. (2007). Human *Streptococcus suis* outbreak in Phayao province, Thailand, 2007. Outbreak Surveill. Investig. Response J..

[20] Wangkaew S., Chaiwarith R., Tharavichitkul P., Supparatpinyo K. (2006). *Streptococcus suis* infection: A series of 41 cases from Chiang Mai University Hospital. J. Infect..

[21] Wangsomboonsiri W., Luksananun T., Saksornchai S., Ketwong K., Sungkanuparph S. (2008). *Streptococcus suis* infection and risk factors for mortality. J. Infect..

[22] Wongsawat S. (2010). *Streptococcus suis* serotype 2 outbreak at Chomthong district, Chiang Mai province, June–July, 2008. Lanna Public Health J..

[23] Tanner N.A., Zhang Y., Evans T.C. (2015). Visual detection of isothermal nucleic acid amplification using pH-sensitive dyes. BioTechniques.

[24] Noppon B., Khaeng S., Sopa A., Phuaram P., Wongsan R., Laohasinnurak T. (2014). *Streptococcus suis* serotype 2 in Uncooked Pork Meat Products in Khon Kaen, Northeastern Thailand, and their Antimicrobial Profiles. Int. J. Sci. Eng. Res..

[25] Lunha K., Chumpol W., Samngamnim S., Jiemsup S., Assavacheep P., Yongkiettrakul S. (2022). Antimicrobial Susceptibility of *Streptococcus suis* Isolated from Diseased Pigs in Thailand, 2018–2020. Antibiotics.

[26] Stanojkovic A., Astojic-andric D., Petrovic M., Stanisic N., Gogic M., Stanojkovic-Sebic A., Radovic C. (2016). Prevalence of *Streptococcus suis* serotype 2 strains isolated from major parts of fresh pork meat. Sci. Works. Ser. C Vet. Med..

[27] Segura M., Aragon V., Brockmeier S.L., Gebhart C., Greeff A., Kerdsin A., O’Dea M.A., Okura M., Saléry M., Schultsz C. (2020). Update on *Streptococcus suis* Research and Prevention in the Era of Antimicrobial Restriction: 4th International Workshop on *S. suis*. Pathogens.

[28] Kerdsin A., Dejsirilert S., Puangpatra P., Sripakdee S., Chumla K., Boonkerd N., Polwichai P., Tanimura S., Takeuchi D., Nakayama T. (2011). Genotypic profile of *Streptococcus suis* serotype 2 and clinical features of infection in humans, Thailand. Emerg. Infect. Dis..

[29] Rayanakorn A., Katip W., Goh B.H., Oberdorfer P., Lee L.H. (2019). Clinical Manifestations and Risk Factors of *Streptococcus suis* Mortality Among Northern Thai Population: Retrospective 13-Year Cohort Study. Infect. Drug Resist..

[30] Rayanakorn A., Goh B.-H., Lee L.-H., Khan T.M., Saokaew S. (2018). Risk factors for *Streptococcus suis* infection: A systematic review and meta-analysis. Sci. Rep..

[31] Lee I.S., Kim W., Jo G., Yang K.Y. (2024). Rapid detection of a downy mildew pathogen, Peronospora destructor, in infected onion tissues and soils by loop-mediated isothermal amplification. Phytopathology.

[32] Gebregziabher S.M., Yalew A.W., Sime H., Abera A. (2024). Molecular detection of waterborne pathogens in infants’ drinking water and their relationship with water quality determinants in eastern Ethiopia: Loop-mediated isothermal amplification (LAMP)-based study. J. Water Health.

[33] Vielba-Fernández A., Dowling M., Schnabel G., Fernández-Ortuño D. (2023). A Loop-Mediated Isothermal Amplification Assay for the Identification of Botrytis fragariae in Strawberry. Plant Dis..

[34] Takabatake R., Kagiya Y., Futo S., Minegishi Y., Soga K., Shibata N., Kondo K. (2023). Rapid Screening Detection of Genetically Modified Papaya by Loop-Mediated Isothermal Amplification. Biol. Pharm. Bull..

[35] Takeuchi D., Kerdsin A., Akeda Y., Chiranairadul P., Loetthong P., Tanburawong N., Areeratana P., Puangmali P., Khamisara K., Pinyo W. (2017). Impact of a Food Safety Campaign on *Streptococcus suis* Infection in Humans in Thailand. Am. J. Trop. Med. Hyg..

[36] Kerdsin A., Segura M., Fittipaldi N., Gottschalk M. (2022). Sociocultural Factors Influencing Human *Streptococcus suis* Disease in Southeast Asia. Foods.

